# Factors influencing high respiratory mortality in coal-mining counties: a repeated cross-sectional study

**DOI:** 10.1186/s12889-019-7858-y

**Published:** 2019-11-08

**Authors:** Ruoding Shi, Susan Meacham, George C. Davis, Wen You, Yu Sun, Cody Goessl

**Affiliations:** 1 Department of Agricultural & Applied Economics, Virginia Tech, 250 Drillfield Drive, Blacksburg, VA 24061 USA; 2 Edward Via College of Osteopathic Medicine, Biomedical Sciences, 2265 Kraft Drive, Blacksburg, VA 24061 USA; 3 Department of Public Health Sciences, University of Virginia, 200 Jeanette Lancaster Way, Charlottesville, VA 22903 USA; 4 China Center for Health Economic Research, Peking University, Beijing, 100871 China; 5 Department of Health Promotion, Social and Behavioral Health, University of Nebraska Medical Center, 42nd and Emile, Omaha, NE 68198 USA

**Keywords:** Coal mining, Respiratory mortality, Appalachia, Health access, Health disparity

## Abstract

**Background:**

Previous studies have associated elevated mortality risk in central Appalachia with coal-mining activities, but few have explored how different non-coal factors influence the association within each county. Consequently, there is a knowledge gap in identifying effective ways to address health disparities in coal-mining counties. To specifically address this knowledge gap, this study estimated the effect of living in a coal-mining county on non-malignant respiratory diseases (NMRD) mortality, and defined this as “coal-county effect.” We also investigated what factors may accentuate or attenuate the coal-county effect.

**Methods:**

An ecological epidemiology protocol was designed to observe the characteristics of three populations and to identify the effects of coal-mining on community health. Records for seven coal-mining counties (*n* = 19,692) were obtained with approvals from the Virginia Department of Health Office of Vital Statistics for the years 2005 to 2012. Also requested were records from three adjacent coal counties (*n* = 10,425) to provide a geographic comparison. For a baseline comparison, records were requested for eleven tobacco-producing counties (*n* = 27,800). We analyzed the association of 57,917 individual mortality records in Virginia with coal-mining county residency, county-level socioeconomic status, health access, behavioral risk factors, and coal production. The development of a two-level hierarchical model allowed the coal-county effect to vary by county-level characteristics. Wald tests detected sets of significant factors explaining the variation of impacts across counties. Furthermore, to illustrate how the model estimations help explain health disparities, two coal-mining county case studies were presented.

**Results:**

The main result revealed that coal-mining county residency increased the probability of dying from NMRD. The coal-county effect was accentuated by surface coal mining, high smoking rates, decreasing health insurance coverage, and a shortage of doctors. In Virginia coal-mining regions, the average coal-county effect increased by 147% (*p*-value< 0.01) when one doctor per 1000 left, and the effect increased by 68% (*p*-value< 0.01) with a 1% reduction of health insurance rates, holding other factors fixed.

**Conclusions:**

This study showed a high mortality risk of NMRD associated with residents living in Virginia coal-mining counties. Our results also revealed the critical role of health access in reducing health disparities related to coal exposure.

## Background

Health disparities have persisted in central Appalachia for decades [1–4]. Virginia mines, in the heart of central Appalachia, in the rugged mountains of the southwestern part of the state, produce high-quality coal. Coal is the heart of the economy and a cultural icon in a region that reveres “coal as king.” While rates of mortality have improved in the region, they have persisted at rates higher than regional and national averages, particularly non-malignant respiratory diseases (NMRD) [3]. Studies attribute the elevated mortality risk to environmental exposure to coal extraction, processing, and transportation activities [5–8]. Mining releases a large amount of coal dust and methane into the environment and results in higher concentrations of particulate matter and sulfate, impairing coal miner’s respiratory system, a condition known as coal workers’ pneumoconiosis (CWP) [9]. Another coal-related lung disease is silicosis caused by inhalation of crystalline silica dust. However, the potential health effects of environmental contaminants produced by coal mining on community residents are the subject of ongoing investigations [10].

The health effects of coal mining are likely to be back in the spotlight of health policymakers as the U.S. government is attempting to revive the coal industry. The U.S. coal production has risen by 4% from 2016 to 2018 [11]. Some are concerned that the reemergence of the coal industry may have negative impacts on the health of those living in these areas, retarding or reversing the progress made to improve health metrics for those residents over the past few decades [12]. For instance, Environmental Protection Agency is proposing to weaken the Coal Ash Regulations to create new coal-related jobs, even though their analysis suggests the new rules will lead to 1400 more premature deaths annually [13]. Black lung disease resurgence for coal miners has been observed in the state of Virginia [14], but the health effect on local communities has not been widely evaluated. The current agenda of bringing back “beautiful clean coal” makes the research into coal’s health impacts on the general population critical [13].

Other factors, such as access to healthcare, could also accentuate or attenuate the adverse effects of coal mining on health. For example, an accentuating factor is noticed when Kentucky lawmakers passed a House bill (HB2-18RS) that permitted fewer doctors to read chest X-ray for miners’ health claims [15]. An example of an attenuating factor was seen when Congress required governments and coal companies to pay out healthcare and guarantee benefits to retired coal workers even as coal companies faced bankruptcy [16]. In these scenarios, the legislative actions are potentially influencing the health of coal community residents.

### Previous literature

Following Meacham et al. [2], we classified studies on health disparities in Appalachia into two groups: those focusing on coal mining and those not focusing on coal-related factors. In the second group, the authors have identified several determinants predominantly associated with health disparities in coal communities, such as low staffing levels in hospitals and Appalachian cultural beliefs [1, 4, 17, 18]. Based on a survey on healthcare providers, Denham et al. [17] found that insufficient health staffing and facilities, and lack of diabetes education explained high diabetes prevalence in Appalachia. This research group also proposed that cultural and ethnic components of communities contributed to poor health outcomes as well. McGarvey et al. [18] suggested a cultural component and revealed that Appalachian residents in Virginia were more likely to report their health status as “poor” compared to non-Appalachian residents even though there was no difference in chronic diseases reported by Appalachian and non-Appalachian groups.

Several studies have focused specifically on coal mining and poor health outcomes in central Appalachia. These poor health outcomes include high mortality rates of cancer [5], cardiovascular diseases [19] and kidney diseases [20], and increased risk of hospitalization for hypertension and chronic obstructive pulmonary disease (COPD) [21]. For instance, Hendryx et al. [7] examined county mortality rates and found that living in a heavy coal-mining county was a risk factor for lung cancer. Based on a telephone survey on the self-reported presence of specific chronic diseases, Hendryx and Ahern [6] tested whether coal production had adverse effects on local residents’ health after controlling demographic characteristics and county-level covariates (smoking rate, obesity rate, poverty rate, and social capital). They found higher risks of cardiopulmonary diseases, chronic lung diseases, hypertension, and kidney diseases were associated with residents living in counties with high-level coal production, compared to residents in non-coal counties.

To identify the health effect of coal mining, most studies have attempted to handle several confounding factors in central Appalachia [3, 7, 19]. However, these health effects have often been assumed constant between coal-mining counties even after controlling socioeconomic and behavioral factors, such as poverty rates and smoking rates [7, 19]. None of the previous studies have considered if the health effects may differ by county and what factors influenced those differences. This means that previous studies implicitly assume that the effect stays constant over time and across other covariates (e.g., coal production, SES, health access). The data availability and limited study scope may have contributed to this literature gap.

### Current approach

For the purposes of this study, the term “*coal-county effect*” has been adopted to refer to the health effect of living in a coal-mining county on mortality.^1^ Using an ecological epidemiology protocol, we estimated the associations between the mortality risk of NMRD and coal-mining county residency and what non-coal factors affect the associations. The non-coal factors of interest represented the geography, temporal trends, and socioeconomic demographics of our study population groups.

Our study objective was twofold, prompting the following research questions:

1. Are the coal-county effects constant across counties?
2. What factors lead to non-constant coal-county effects?

With the first question, we hypothesize that the coal-county effect may depend on a county’s health access, economic condition, coal production, and other health behavioral risk factors.^2^ For example, limited access to health care services could accentuate coal-county health effects, because some coal-related lung diseases (e.g., CWP and silicosis) are often symptomless in the early stages but develop into severe conditions without access to screening services and treatments [22]. By addressing the second question, we plan to identify and estimate the impact of selected factors contributing to the existing poor health measures in coal counties. The development of a novel, two-level hierarchical model allows the estimated coal-county effect to vary depending on the county’s socioeconomic status, health access, health behavioral risk factors and coal production. Following the insights from Hendryx et al. [7] and Hendryx et al. [23], we consider coal production from both surface mining (i.e., strip mining, open-pit mining, and mountaintop removal mining) and underground mining. Surface mining practice is more likely to affect neighboring communities by air and water pollution [10], while underground coal mining is often associated with miners’ lung diseases, an occupational hazard [22].

## Methods

### Study design

Individual death records (*n* = 57,917) were merged with county-level covariates based on their counties of residence and years of death to capture the dynamic changes from 2005 to 2012. Ethical approvals of individual mortality data were obtained from the Internal Review Boards of the Edward Via College of Osteopathic Medicine and the Virginia Department of Health Office of Vital Statistics. County-level covariates were selected to capture the multi-dimensional concepts of socioeconomic status, health access, and health behavioral risk factors in three population subgroups. Our model design allowed the coal-county effect to vary as a function of selected county-level covariates. This model framework enabled us to test the assumption of non-constant coal-county health effects. It also identified factors that explain variations across coal counties.

This study considered a potential spillover effect of coal production across county borders, which had not been explored in the majority of previous research using non-mining counties as reference groups. The spatial analysis by Hitt and Hendryx [24] showed that cancer mortality rates were autocorrelated between adjacent counties. Although our analysis was not of the typical spatial approach, we did analyze the counties adjacent to coal-mining counties to test a spillover effect. We considered both coal-mining counties and counties adjacent to coal-mining counties as “treated” groups. Since Virginia tobacco counties share similar economic characteristics with coal-mining counties, such as “low economic diversification, low employment in professional services, and low educational attainment rates [25]”, these tobacco counties served as a control group or “untreated” baseline counties. Then, we identified the coal-county effect by comparing the average likelihood of dying from NMRD among residents in treated groups with that in baseline counties. The choice of an “untreated” baseline aimed to reduce selection bias because of the similarity between coal-mining counties and tobacco counties.

### Study area

With places of residence recorded, the mortality data were collected from three rural, underserved health disparity areas in Virginia: coal-mining counties, adjacent coal counties, and tobacco counties. The adjacent coal counties served as a geographic comparison group with residents living in small communities in mountainous southwest Virginia. The tobacco counties were an economic comparison group located in south central Virginia and experienced financial trends over several decades that were similar to those for coal-dependent counties.

**Fig. 1:**
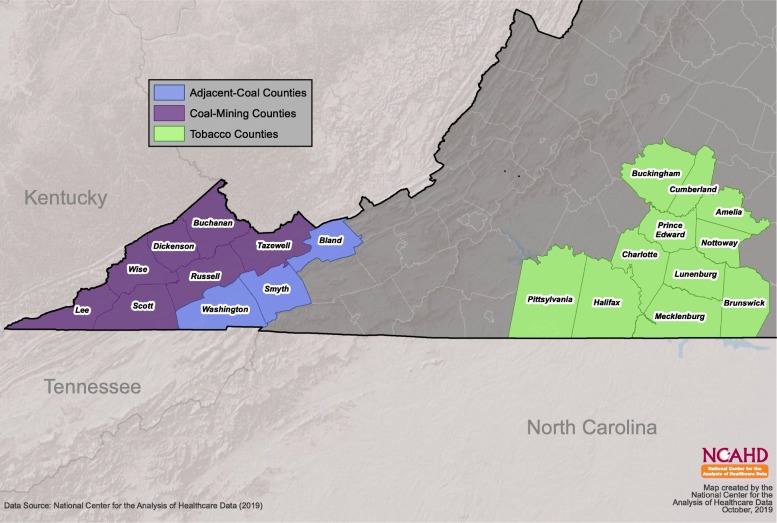
Study area by three county groups in Virginia. *Source: National Center for the Analysis of Healthcare Data. Permission has been obtained to publish this figure.*

Figure 1 shows the three county groups in Virginia. Seven counties in southwest Virginia were considered as coal-mining counties (Buchanan, Dickenson, Lee, Russell, Scott, Tazewell, and Wise, *n* = 19,692 records). Although Scott County stopped producing coal after 1995, it was classified as a coal-mining county because coal mining may have a long-run impact on the local environment and human health, particularly chronic conditions [26]. When estimating the coal-county effect, we run alternative models in which Scott County is treated as an adjacent coal county to check if the results are sensitive to this classification. Three Virginia counties share the county border with coal-mining counties (Bland, Smyth and Washington, *n* = 10,425 records). The 11 tobacco counties are located in the region historically known for tobacco production (Amelia, Brunswick, Buckingham, Charlotte, Cumberland, Halifax, Lunenburg, Mecklenburg, Nottoway, Pittsylvania, and Prince Edward, *n* = 27,800 records). These counties dependent on tobacco industry as a primary source of the local economy and are economically comparable to coal-mining counties [2]. Therefore, we used them as baseline counties.

### Data sources and variables

#### Individual-level data

Death records were collected from the Virginia Department of Health Office of Vital Statistics [27], included the primary cause of death, age, gender, place of residence, marital status, and years of education. Our outcome variable was death caused by non-malignant diseases of the respiratory system with the International Codes for Diseases (ICD) 10th revision codes J00 – J99. NMRD includes but is not limited to asthma, chronic obstructive pulmonary disease (COPD), and the pneumoconiosis. NMRD was chosen as the dependent variable of concern because this group of diseases was commonly considered as a high-risk health problem in coal-mining regions [3, 23]. For example, counties in central Appalachia had the highest mortality rates of pneumoconiosis and COPD [28].

#### County-level covariates

Publicly available county annual coal production was obtained from the U.S. Energy Information Administration [29]. Other county-level covariates were collected from multiple sources and classified into three categories: socioeconomic characteristics, accessibility of health care services, and health behavioral risk factors. Most county-level covariates were obtained from the Area Health Resources File (AHRF) [30]. AHRF is a health resource information system maintained by the Health Resources and Services Administration. County health behavioral risk factors were obtained from the Behavioral Risk Factor Surveillance System (BRFSS) data [31]. Finally, additional data sources included the Census Bureau’s Small Area Health Insurance Estimates (SAHIE) for health insurance rates and the U.S. Census Bureau and Rural-Urban Continuum code from the United States Department of Agriculture Economic Resource Service.

Selected covariates included county unemployment rates, median household income, and rural-urban status to measure SES, which played a vital role in individuals’ health outcomes and likelihoods of dying. The first SES variable was the unemployment rate at the county level as unemployment increased mortality risk by keeping jobless people from investing in health [32]. However, employment alone was insufficient to measure available resources since a majority of individuals in the sample were retired. We also considered county median household income and unobserved differences between rural and urban residents. Based on the nearest observed Rural-Urban Continuum codes in 2003 and 2013, we constructed indicators to classify counties into rural counties, non-metropolitan urban counties, and counties in the metropolitan area.^3^ The 2003 Rural-Urban Continuum codes were used to construct indicators starting in 2005 due to a closer time reference, and then we switched to 2013 Rural-Urban Continuum codes to classify counties after 2008.

To represent health access, county health insurance rates were collected from SAHIE and three county-level health access measurements from AHRF, including numbers of doctors (sum of active medical doctors and osteopathic doctors), hospital beds and health centers per 1000 population. Finally, we collected smoking rates at the county level from the study of Dwyer-Lindgren et al. [33] and age-adjusted obesity rates and physical inactivity prevalence rates from the BRFSS.

### Empirical model

A two-level latent index model was used to estimate the coal-county effect and adjacent-coal-county effect [34]. A multilevel modeling technique is one type of regression analysis that handles micro-level individual and macro-level county factors simultaneously in one model [35]. In the context of this study, traditional regression approaches do not consider between-county heterogeneity and assume the coal-county effect is constant across all coal counties. A less restricted assumption is that the statistical association between coal mining and health outcome follows a distribution, and it can be different across coal counties and over time due to other covariates, such as SES or health behavioral risk factors. Instead of fitting a different model based on each county’s individual-level data, we used a two-level model with individuals (level 1) nested within counties (level 2) and allowed key model parameters vary across counties and over time in association with other covariates. A detailed description of each level’s model specification was provided below.

The *Level-1 Model* assumes that for an individual *i* in county *j* deceased in year *t*, the probability of dying from a certain disease *y*_*ijt*_ could be estimated through a latent index ${y}_{ijt}^{\ast }$. Intuitively, the latent index ${y}^{\ast }$ reflects the severity of a disease: the individual will die when the latent index reaches a threshold (${y}^{\ast }$ > 0). We model the latent index as a linear combination of county-specific intercept (*β*_0*jt*_), county group indicator (coal-mining, adjacent coal or tobacco county), individual ’s demographic characteristics (***X***_***ijt***_) and year-specific effects (***d***_***t***_) as follows:

1 $$ {y}_{ijt}^{\ast }={\beta}_{0 jt}+{c}_{1 jt}{d}_{incoal}+{c}_{2 jt}{d}_{adjcoal}+{\boldsymbol{X}}_{\boldsymbol{ijt}}^{\prime }{\boldsymbol{\beta}}_{\mathbf{1}}+{\boldsymbol{d}}_{\boldsymbol{t}}^{\prime}\boldsymbol{\sigma} +{\varepsilon}_{ijt} $$

To estimate the coal-county effect and adjacent-coal-county effect, we use two binary variables indicating county groups: *d*_*incoal*_ = 1 if the deceased lived in a coal-mining county, and *d*_*adjcoal*_ = 1 if the deceased lived in a county adjacent to coal-mining counties. The baseline group consists of those residing in tobacco-producing counties due to the similarity between coal-mining counties (“treated” group) and tobacco-producing counties (“untreated” group) and their non-adjacency. Additionally, individual-level demographic variables (***X***_***ijt***_), such as age, race and gender are included. A set of year dummies (***d***_***t***_) is added to control unobserved time effects. We adjust errors (*ε*_*ijt*_) for correlations between individuals in the same county.

The *Level-1 Model* (1) allows three parameters to vary by county *j* and year *t*: *β*_0*jt*_, *c*_1*jt*_, and *c*_2*jt*_. The parameter *β*_0*jt*_ is the tobacco-county-specific intercept, reflecting county heterogeneity in the mean latent index at the baseline when *d*_*incoal*_ = *d*_*adjcoal*_ = 0. We call *β*_0*jt*_ the “*county baseline”* as a short term in the following discussion since tobacco counties are chosen as baseline counties. As the probability of dying from a specific disease is an increasing function of the latent index, a lower county baseline suggests a lower mean county probability of dying. We expect the county baseline (i.e., free of coal mining effect) to be lower if that county’s residents have a higher socio-economic status (SES), better health access (HA) and lower health behavioral risk (HR) at year *t*. Suppose there are two counties, and county A provides better health access than county B. This expectation can be explained in two scenarios. 1) When residents in both counties are the same in SES and HR aspects, residents in county A would be less likely to die from a particular disease; 2) When residents in both counties also differ in some of SES and HR aspects, for instance, if residents in county A face a higher unemployment rate and smoking rate compared to residents in county B, this may offset their advantage with health access and result in a higher likelihood of dying. Therefore, the county baseline is determined by the specific combination of SES, HA, and HR. We further specify *β*_0*jt*_ in the *Level-2 Model* with the signs indicating prior expectations.

2 $$ \underset{\left(?\right)}{\beta_{0 jt}}=\underset{\left(?\right)}{\beta_0}+\underset{\left(-\right)}{\eta_{01}} SE{S}_{jt}+\underset{\left(-\right)}{\eta_{02}}H{A}_{jt}+\underset{\left(+\right)}{\eta_{03}}H{R}_{jt} $$

The level-2 predictors *SES*, *HA,* and *HR* are a set of county characteristics that could affect the intercept, as introduced in the section of County-level covariates.

The parameter *c*_1*jt*_ measures the average coal-county effect by comparing mean latent indices between a coal-mining county with a tobacco county, holding other factors fixed. We expect *c*_1*jt*_ > 0 if living in a coal-mining county contributed to the mortality risk. Like *β*_0*jt*_, we hypothesize the coal-county effect to be different among coal-mining counties. In addition to SES, health access and health behavioral risk factors, total coal production (*Prod*) and the percent of production from surface coal mining (*Surface*%), may have also affected the link between coal production and mortality risks. Consequently, similar to *β*_0*jt*_, the coefficient *c*_1*jt*_ is allowed to vary by county characteristics.

3 $$ \underset{\left(+\right)}{c_{1 jt}}=\underset{\left(?\right)}{c_1}+\underset{\left(-\right)}{\eta_{11}} SE{S}_{jt}+\underset{\left(-\right)}{\eta_{12}}H{A}_{jt}+\underset{\left(+\right)}{\eta_{13}}H{R}_{jt}+\underset{\left(+\right)}{\eta_{14} Pro{d}_{jt}}+\underset{\left(+\right)}{\eta_{15} Surface{\%}_{jt}} $$

The magnitude of coal-county effect (*c*_1*jt*_) depends on estimated parameters and historical values of county characteristics, which change over year (*t*) and differ for each county (*j*). Therefore, *c*_1*jt*_ is heterogeneous both within and between counties. To explain this intuitively, we expect coal-mining county *j* ’s adverse health effect could be reduced over time if county *j* improves the economic status of residents, increases the accessibility of health care services or decreases risk factors and coal production (within-county heterogeneity). Also, a coal-county effect is expected to be smaller for a coal-mining county with higher SES, better *HA*, lower *HR* and coal production and less surface mining activities, compared with other coal-mining counties during the same year *t* (between-county heterogeneity). Note, although *η*_11_ and *η*_12_ are expected to be negative, *c*_1*jt*_ could still be positive if the effects of the health behavioral risk factors (HR), coal production (*Prod*) and surface coal percentage (*Surface*%) offset the socioeconomic status (SES) and health access (HA) effects.

Similar logic applies to adjacent coal counties, so the adjacent-coal-county effect is specified as:

4 $$ \underset{\left(+\right)}{c_{2 jt}}=\underset{\left(?\right)}{c_2}+\underset{\left(-\right)}{\eta_{21}} SE{S}_{jt}+\underset{\left(-\right)}{\eta_{22}}H{A}_{jt}+\underset{\left(+\right)}{\eta_{23}}H{R}_{jt} $$

If some coal mines are located near the county boundaries, *c*_2*jt*_ is expected to be positive. Again, *η*_21_ and *η*_22_ are expected to have negative signs, indicating higher SES and better health access reducing the adjacent-coal-county effect on mortality. Since health behavioral risk factors increase the county effect [36], *η*_23_ is expected to be positive.

Substituting Eqs. (2) to (4) into Eq. (1), yields:

5 $$ {\displaystyle \begin{array}{cc}& {y}_{ijt}^{\ast }=\left({\beta}_0+{\eta}_{01} SE{S}_{jt}+{\eta}_{02}H{A}_{jt}+{\eta}_{03}H{R}_{jt}\right)+\Big({c}_1\\ {}& +{\eta}_{11} SE{S}_{jt}+{\eta}_{12}H{A}_{jt}+{\eta}_{13}H{R}_{jt}+{\eta}_{14} Pro{d}_{jt}+{\eta}_{15} Surface{\%}_{jt}\left){d}_{incoal}+\right({c}_2\\ {}& +{\eta}_{21} SE{S}_{jt}+{\eta}_{22}H{A}_{jt}+{\eta}_{23}H{R}_{jt}\Big){d}_{adjcoal}+{\boldsymbol{X}}_{\boldsymbol{ijt}}^{\prime }{\boldsymbol{\beta}}_{\mathbf{1}}+{\boldsymbol{d}}_{\boldsymbol{t}}^{\prime}\boldsymbol{\sigma} +{\varepsilon}_{ijt}\end{array}} $$

To answer the research questions, we test the following two hypotheses:

1. Parameters *β*_0*jt*_, *c*_1*jt*_ and *c*_2*jt*_ vary between counties and over time. This means *η*_01_, *η*_02_ and *η*_03_ are not jointly equal to zero in the intercept equation. The same logic is applied to *c*_1*jt*_ equation (*η*_11_ ≠ 0 or *η*_12_ ≠ 0 or *η*_13_ ≠ 0 or *η*_14_ ≠ 0 or *η*_15_ ≠ 0) and *c*_2*jt*_ equation (*η*_21_ ≠ 0 or *η*_22_ ≠ 0 or *η*_23_ ≠ 0);
2. The coal-county effect is affected by socioeconomic status, health access, high-risk behavioral factors, and coal production. This means the coefficients *η*_11_ ≠ 0, *η*_12_ ≠ 0, *η*_13_ ≠ 0, *η*_14_ ≠ 0 and *η*_15_ ≠ 0 in Eq. (3).

### Statistical analyses

Our statistical analyses began with a descriptive summary of all variables in the model. In order to test for the first hypothesis, we estimated the general model specified by Eq. (5) with all explanatory variables. Wald tests were conducted to test the joint significance of all county-level covariates in the *β*_0*jt*_, *c*_1*jt*_, and *c*_2*jt*_ equations. The model assumed that individuals were correlated within the same counties or cities. According to Cameron and Miller [37], ordinary Wald tests often over-reject when there is a small number of counties (M = 24 clusters in our case^4^), meaning that the *p*-values from ordinary Wald tests are underestimated. We followed their suggestion and conducted adjusted Wald tests, which were based on a t-distribution with M-1 degrees of freedom. All statistical analyses were conducted using Stata 14 software [38].

For the second hypothesis, particular interest centered on the coal-county effect *c*_1*jt*_ in Eq. (3). According to the Wald tests, we adjusted the general model by excluding non-significant vectors of variables and checked sensitivities of the results to different specifications. Variance inflation factor (VIF) was used to test potential collinearity between socioeconomic and health access covariates. Next, the coal-county effects (*c*_1*jt*_) of three coal-mining counties were predicted based on these counties’ historical characteristics and the estimated parameters. The case study of two Virginia coal-mining counties (Russell County and Lee County) illustrated how our finding could be meaningful in the real world. Specifically, it explained what happened to the coal-county effect when some non-coal factor changed over time.

Although our analyses were not able to identify coal miners from the death records, we expected that male and working-age residents in our sample would have a higher mortality risk associated with coal mining, because this population would more likely to be working in coal mines. To explore this, we ran the regressions and predicted the coal-county effects for male and female subgroups separately. Similar analyses were also conducted on working age (15–64) and retirement age (> 64) subgroups.

## Results

### Descriptive statistics

Table 1 provides descriptive statistics for all variables at the individual level (*n* = 57,917). From 2005 to 2012, an average of 11 out of 100 people died from NMRD. Residents in the death records obtained an average of 10 years of schooling (standard deviation (SD) = 3.56), and their average age was 72 years (SD = 17.55). The majority of deceased individuals were white (83%), and one half of the sample was female. About 39% of the deceased were married. Consistent with previous literature, SES in this region was relatively low. The average county unemployment rate was 7% (SD = 2%), and the mean of median household income was $35,880 (SD = 4120). About 39% of residents lived in rural areas where the population was less than 2500. On average, the age-adjusted physical inactivity prevalence rate was 28% (SD = 3%), and the age-adjusted obesity rate was 30% (SD = 3%). The average smoking rate of 28% (SD = 2%) was above the national average of around 24% calculated by Dwyer-Lindgren et al. [33]. Regarding health access variables, the mean values of hospital beds, federal qualified health centers and doctors were 3.08, 0.06 and 1.11 per 1000 population, respectively. The average county health insurance rate showed that 84% of individuals had some sorts of health insurance. Among the study area, the mean county annual coal production was 1.23 million tons with a large standard deviation of 2.87, which indicated heterogeneity in coal production between counties. Except for Scott County, all coal-mining counties in Virginia were involved in surface mining, and the mean surface coal production was 0.52 million tons (SD = 1.37). Finally, of the 57,917 residents in the death records, 19,692 residents (34%) were living in seven coal-mining counties and 10,425 residents (18%) in three adjacent counties.

**Table 1:** Summary of individual and county-level characteristics from years 2005 to 2012. (*n* = 57,917)

Variable	Definition and Label	Mean	SD ^a^	Min ^b^	Max ^c^
Dependent variable					
*Y*_*NMRD*_	Death indicator: 1 = Death due to Non-Malignant Respiratory Disease	0.11	0.32	0	17
Demographics					
*edu*	Years of education	10.08	3.56	0	17
*ag*e	Age in years	72.24	17.55	0	109
*I*_*white*_	Race indicator: 1 = white	0.83	0.38	0	1
*I*_*black*_	Race indicator: 1 = black	0.17	0.38	0	1
*I*_*othe*r_	Race indicator: 1 = other race except for white and black	0.002	0.05	0	1
*female*	Gender indicator: 1 = female	0.50	0.50	0	1
*I*_*singl*e_	Marital status indicator: 1 = single	0.11	0.31	0	1
*I*_*marrie*d_	Marital status indicator: 1 = married	0.39	0.49	0	1
*I*_*widowed*_	Marital status indicator: 1 = widowed	0.38	0.48	0	1
*I*_*divor*_	Marital status indicator: 1 = divorced	0.13	0.33	0	1
SES					
*R*_*unemploy*_	Unemployment rate	0.07	0.02	0.03	0.1
*Income*	Median household income in 1000 dollars	35.88	4.12	25.2	52.4
*I*_*metro*_	Rural-urban indicator: 1 = county in a metro area	0.28	0.45	0	1
*I*_*nonmetro*_	Rural-urban indicator: 1 = nonmetropolitan county with the urban population more than 2500	0.33	0.47	0	1
*I*_*rural*_	Rural-urban indicator: 1 = nonmetropolitan county completely rural with less than 2500	0.39	0.49	0	1
Risk factors					
*R*_*inactivity*_	Age-adjusted leisure-time physical inactivity prevalence percent	0.28	0.03	0.2	0.4
*R*_*obesity*_	Age-adjusted obesity rate	0.30	0.03	0.2	0.4
*R*_*smoking*_	Age-standardized total cigarette smoking prevalence rate	0.28	0.02	0.2	0.3
Health access					
*bed*_*per*1000_	Hospital beds per 1000 population	3.08	3.85	0	15.1
*hcenter*_*per*1000_	Federal qualified health centers per 1000 population	0.06	0.07	0	0.3
*doctor*_*per*1000_	M.D. and D.O. total active non-Fed & fed per 1000 population	1.11	0.84	0.1	2.9
*R*_*insur*_	Percent insured under 65 years (%)	83.70	1.79	78.9	88.2
*d*_>2007_^*d*^	Switch indicator: 1 = year after 2007	0.62	0.49	0	1
Coal-related					
*Prod*	County coal production (million tons)	1.23	2.87	0	11.8
*Surface*	County surface coal production (million tons)	0.52	1.37	0	6.7
*Surface*%	Percent of surface mining coal (%)	11.35	20.66	0	71.7
*d*_*incoal*_	Coal indicator: 1 = live with living in a coal-mining county	0.34	0.47	0	1
*d*_*adjcoal*_	Coal indicator: 1 = living in an adjacent county of coal-mining counties	0.18	0.38	0	1

^*a*^ the SD denotes the standard deviation

^b^ Min denotes the minimum values of each variable

^c^ Max denotes the maximum values of each variable

^d^ The SAHIE program calculates county-level health insurance based on national survey data. In 2008, the SAHIE program switched from using Current Population Survey (CPS) as the basis of estimation to American Community Survey (ACS). Therefore, to capture the structural change of this variable in the model, we add a product of the insurance rate with a switch indicator *d*_>2007_, which is one after 2007

### Wald test results

Table 2 reports *p*-values from adjusted Wald tests (*p*-values from ordinary Wald tests are reported in the parentheses). The first row suggests that varying specifications of *β*_0*jt*_, *c*_1*jt*_, and *c*_2*jt*_ were preferred. For example, in the *c*_1*jt*_ column of row (1), we tested the null hypothesis *H*_0_ : *η*_11_ = *η*_12_ = *η*_13_ = *η*_14_ = *η*_15_ = 0 in Eq. (3) and obtained a p-value less than 0.01 from adjusted Wald test, so we rejected the null hypothesis that the coal-county effect was a constant and independent of county-level covariates. Likewise, Wald tests also rejected the null hypothesis that *β*_0*jt*_ (*p*-value< 0.01) and *c*_2*jt*_ (*p*-value< 0.01) were constants.

**Table 2:** Wald test of varying parameters

Vector of Variables to Test for Joint Insignificance	p-value: adjusted (unadjusted)	*β*_0*jt*_ (1)	*c*_1*jt*_ (2)	*c*_2*jt*_ (3)	*β*_0*jt*_ + *c*_1*jt*_ (4)	*β*_0*jt*_ + *c*_2*jt*_ (5)	*c*_1*jt*_ + *c*_2*jt*_ (6)	*β*_0*jt*_ + *c*_1*jt*_ + *c*_2*jt*_ (7)
(1)All variables except for the intercept		< 0.01 (< 0.01)	< 0.01 (< 0.01)	< 0.01 (< 0.01)	0.02 (< 0.01)	0.02 (< 0.01)	0.14 (< 0.01)	0.29 (< 0.01)
(2) *SES***:** *R*_*unemploy*_, *Income*, *I*_*rural*_, *I*_*metro*_		0.31 (0.21)	0.11 (0.04)	0.01 (< 0.01)	0.01 (< 0.01)	0.04 (< 0.01)	0.02 (< 0.01)	0.06 (< 0.01)
(3) *HA***:** *bed*_*per*1000_, *hcenter*_*per*1000_, *doctor*_*per*1000_, *R*_*insur*_, *R*_*insur*_ ∗ *d*_>2007_		0.08 (0.01)	< 0.01 (< 0.01)	< 0.01 (0.02)	< 0.01 (< 0.01)	< 0.01 (< 0.01)	< 0.01(< 0.01)	< 0.01 (< 0.01)
(4) *HR***:** *R*_*obesity*_***, **** R*_*inactivity*_, *R*_*smoking*_		0.06 (0.02)	0.15 (0.09)	0.61 (0.56)	0.01 (< 0.01)	0.30 (0.12)	0.30 (0.12)	0.08 (< 0.01)

Furthermore, we tested the joint significance of socio-economic status, health access and health behavioral risk vectors of variables separately in each level-2 equation. Column (2) and (3) in Table 2 show that coal-county effect *c*_1*jt*_ and adjacent-coal-county effect *c*_2*jt*_ were significantly affected by health access (HA) with *p*-values less than 0.01, and county SES also explained the variations in adjacent-coal-county effects (p-value = 0.01). The county baseline *β*_0*jt*_ appeared to depend on health access (HA) and health behavioral risk factors (HR) with p-values less than 10%.

### Model results of coal-county effects

Results of collinearity test are provided in Additional file 1. The maximum VIF value was less than 3, which indicated that there was no collinearity. Average marginal effects of all variables are reported in Additional file 2. The average marginal effect of the coal-county indicator was significantly positive across models.

Table 3 reports estimated coefficients in the equation of *c*_1*jt*_, utilizing different model specifications. The magnitude and significance of estimated coefficients were robust. The results show that the coal-county effect was higher in rural and metropolitan urban areas compared to non-metropolitan urban areas. Significant coefficients were found for the number of hospital beds, doctors per 1000 population and health insurance rates. For example, one additional doctor per 1000 population significantly reduced the coal-county effect by 0.119 to 0.147 across models, and a 1% increase in health insurance coverage rates significantly reduced the health effect by 0.065 to 0.070 across models. However, the coefficient of hospital beds per 1000 population is significantly positive. Regarding health behavioral risk factors, a 1% increase of the smoking rate at the county level significantly increased the coal-county health effect by 0.026 to 0.035 across models. Finally, the coal-county effect went up by 0.02 to 0.04 with a 10% increase in surface coal proportion. The coefficients of total coal production were not significant, so this variable was excluded from the final estimation due to high collinearity with surface coal percentage.

**Table 3:** Estimated coefficients of varying coal-county effects

	(1)	(2)	(3)	(4)	(5)
	General Model^a^	Model 1^b^	Model 2^c^	City Adjusted Model^d^	Scott Check Model^e^
Intercept (*c*_1_)	4.134^**^	4.969^***^	5.028^***^	5.028^***^	5.088^***^
	(2.39)	(3.13)	(3.59)	(3.67)	(3.60)
SES					
*R*_*unemploy*_	0.020	0.020			
	(1.09)	(1.24)			
*Income*	0.011	0.011^*^			
	(1.16)	(1.78)			
*I*_*metro*_	0.146^**^	0.092^*^			
	(2.43)	(1.88)			
*I*_*rural*_	0.107^*^	0.079^**^			
	(1.80)	(2.22)			
Health Access					
*bed*_*per*1000_	0.0412^***^	0.041^***^	0.034^***^	0.034^***^	0.031^***^
	(8.57)	(8.71)	(7.76)	(8.38)	(8.09)
*hcenter*_*per*1000_	−0.131	−0.157	−0.140	−0.140	−0.138
	(−0.52)	(− 0.75)	(− 0.64)	(− 0.87)	(− 0.67)
*doctor*_*per*1000_	− 0.119^***^	− 0.143^***^	− 0.147^***^	− 0.147^***^	− 0.141^***^
	(−3.02)	(−4.25)	(−5.92)	(−8.04)	(−5.79)
*R*_*insur*_	− 0.065^***^	− 0.070^**^	− 0.068^***^	−0.068^***^	− 0.065^***^
	(− 3.84)	(− 4.12)	(− 4.08)	(− 4.15)	(− 3.73)
*R*_*insur*_ ∗ *d*_>2007_	− 0.001^*^	− 0.002^*^	− 0.001^*^	−0.001^*^	− 0.001
	(−1.88)	(−2.76)	(−1.84)	(−1.78)	(−1.16)
Risk Factor					
*R*_*obesity*_	0.005	−0.002	0.010	0.010	−0.03
	(0.38)	(−0.19)	(1.13)	(1.09)	(−0.37)
*R*_*inactivity*_	−0.008	−0.006	− 0.005	−0.005	− 0.003
	(−0.88)	(− 0.73)	(− 0.71)	(−0.72)	(− 0.48)
*R*_*smoking*_	0.035^**^	0.029^**^	0.026^***^	0.026^**^	0.027^***^
	(2.52)	(1.97)	(2.64)	(2.45)	(2.71)
Coal Production					
Surface%	0.004^***^	0.003^***^	0.002^***^	0.002^***^	0.002^***^
	(9.14)	(9.72)	(4.68)	(4.71)	(4.55)
Pseudo *R*^2^	0.0297	0.0296	0.0294	0.0294	0.0293
Log likelihood	−16,917	−16,918	−16,921	−16,921	−16,922
BIC	34,103.3	34,085.3	34,100.8	34,068.4	34,104.1
Number of observations	49,437	49,437	49,437	49,437	49,437

z test statistic in parentheses ^*^ *p* < .1, ^**^ *p* < .05, ^***^ *p* < .01

^a^ General model: kept all vectors of SES, HA and HR variables in *β*_0*jt*_, *c*_1*jt*_ and *c*_2*jt*_ in Eq. (5) as the preliminary model

^b^ Model 1: removed all SES variables in *the β*_0*jt*_ equation and all HR variables in the *c*_2*jt*_ equation from the General model

^c^ Model 2: removed all SES variables in both *β*_0*jt*_ and *c*_1*jt*_ equations and all HR variables in the *c*_2*jt*_ equation from the General model

^d^ City adjusted model: since there are independent cities that nest into counties in Virginia, we collapsed these cities into their belonging counties and adjusted the clustered structure of error terms in model 2 accordingly

^e^ Scott check model: provided that Scott County stop producing coal in 1996, we treated Scott County as an adjacent coal county instead of a coal-mining county to check sensitivity using model 2’s specification

### Case studies

**Fig. 2:**
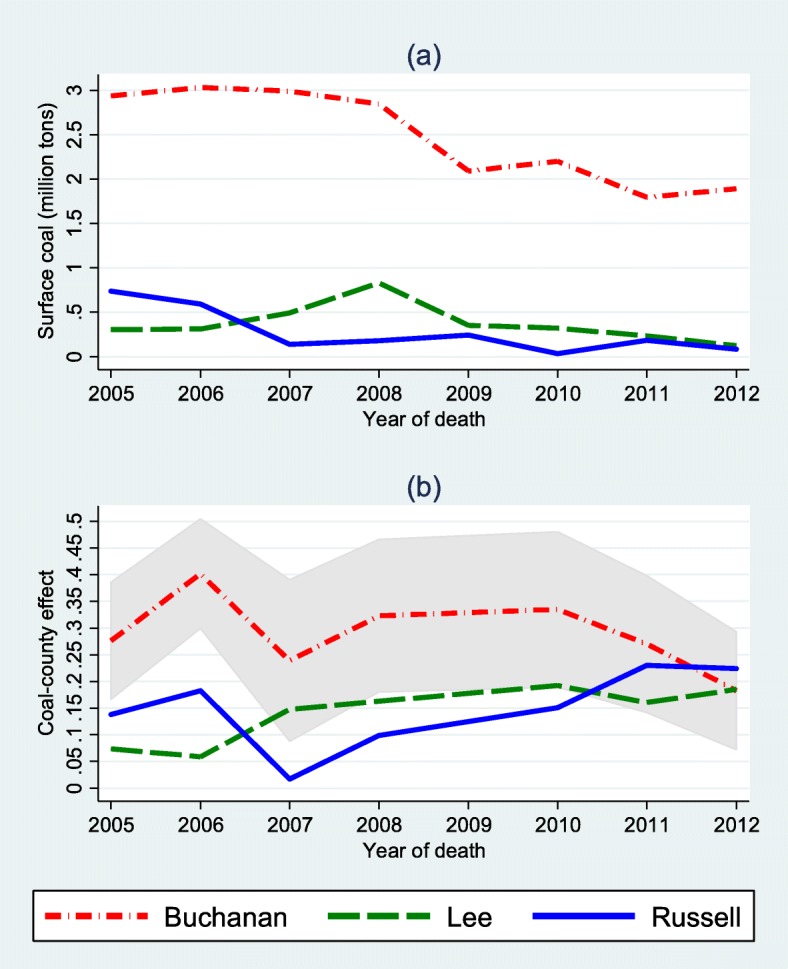
(**a**) Annual surface coal production and (**b**) Predicted coal-county effects of three Virginia coal-mining counties

Figure 2a plots annual surface-mining coal production of three counties in Virginia. Buchanan County had produced the most coal in Virginia in the past decades, and its production started to decline after 2007. Surface coal production in Russell County and Lee County had been much lower and less than 1 million tons. The coal-county effect (*c*_1*jt*_) was predicted using the estimated parameters from model 2 preferred by the adjusted Wald tests. Figure 2b shows the predicted coal-county effects for these three counties: Buchanan County (${\hat{c}}_{1 jt}$: 0.18 to 0.40), Russell County (${\hat{c}}_{1 jt}$: 0.02 to 0.23) and Lee County (${\hat{c}}_{1 jt}$: 0.06 to 0.2). A 95% confidence interval was drawn around Buchanan County’s ${\hat{c}}_{1 jt}$ to indicate the precision of predicted values. The overall average coal-county effects in the Virginia coal region was 0.1 from 2005 to 2012. Highest coal-county effects were observed in Buchanan County because of its heavy coal production. However, the coal-county effects increased rapidly in Russell County and Lee County, although their surface coal production had been flat or decreasing.

**Fig. 3:**
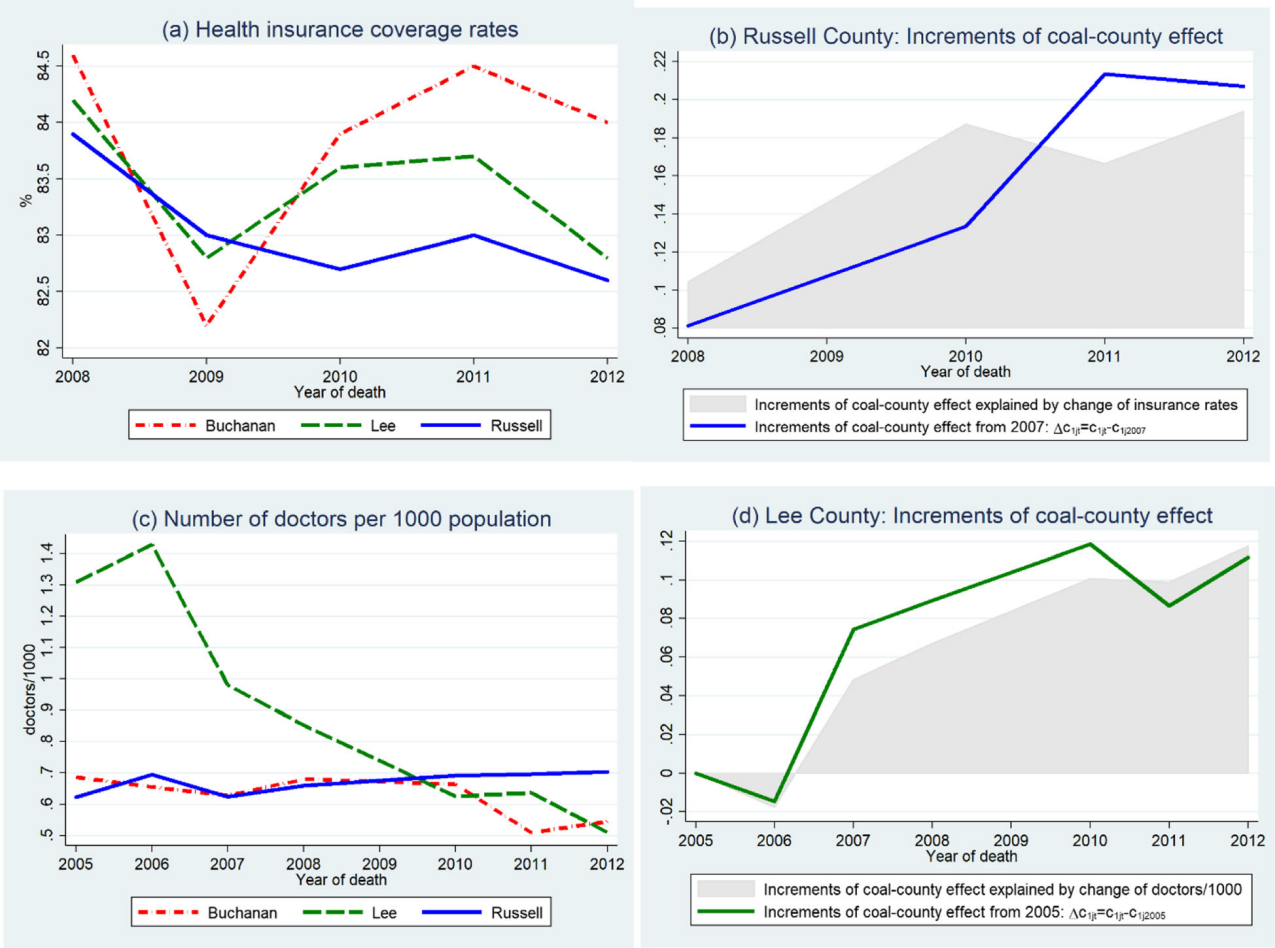
Increasing coal-county effects in two counties caused by deterioration in access to healthcare. (**a**) Health insurance coverage rates, (**b**) Russell County: Increments of coal-county effect, (**c**) Number of doctors per 1000 population and (**d**) Lee County: Increments of coal-county effect. *Note: Year-to-year comparisons of insurance rates are only appropriate after 2007 because the SAHIE program switched the data source in 2008*

Figure 3 provides an intuitive explanation to the increasing coal effect in Russell county. Russell County’s health insurance rates were declining and much lower than other coal-mining counties (Fig. 3a). By plotting the increments of Russell County’s coal effects from 2007 and the fraction of increments explained by health insurance rate (shadow area). Figure 3b shows that Russell County’s declining health insurance rates mainly drove the increasing coal-county effect. Given an average of population of 28,834, our model predicted that a 1% decrease in the health insurance rate would lead to 403 residents dying from NMRD in Russell County, and increase the average coal-county effect by 68%.

Figure 3c shows that doctors were leaving Lee County from 2006, and the decreasing number of doctors explained more than two-thirds of the increments of coal-county effects in Fig. 3d. Model result suggested that the average coal-county effect increased by 147% (=0.147/0.1*100%) with one additional doctor per 1000 population leaving.

### Subgroup analyses

**Fig. 4:**
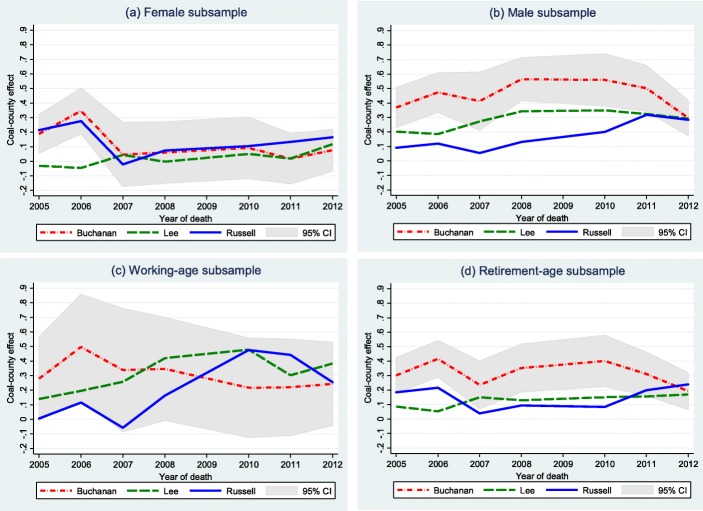
Subsample predicted coal-county effects (**a**) Female, (**b**) Male, (**c**) Working-age and (**d**) Retirement-age

Figure 4a and b show the predicted coal-county effects from the female-only model and male-only model under the specification of model 2. The predicted coal-county effects on females ranged between 0 to 0.1 since 2007, and the marginal effect of coal-county indicator was not significant. However, for males, we found that the coal-county effects ranged between 0.1 to 0.5, and coal-mining county residency significantly increased the probability of dying from NMRD.

Next, Fig. 4c and d show the predicted coal-county effects for working age (15–64) and retirement age (> 64), respectively. With an average of 0.18, the coal-county effects were stronger for the working-age population, while the average coal-county effect on the retirement-age population was 0.10. Since the working-age sample had a smaller sample size than other subsamples, the width of its 95% confidence interval was around 0.6, while other confidence intervals’ width was around 0.2 to 0.3. For Russell County, a 0.5 increase in coal-county effect was observed for the working population after 2007 in Fig. 4c, but not for the retirement-age population in Fig. 4d.

## Discussion

The positive marginal effect of the coal county indicator indicated that, compared to a tobacco county, living in a coal county increased the probability of dying from NMRD. Although residents in adjacent coal counties were exposed to similar pollutants from coal production, we did not find higher mortality risk associated with residence in an adjacent-coal county. Additionally, several non-coal factors (i.e., health insurance coverage rates, numbers of doctors and hospital beds and smoking rates) significantly affected the coal-county effect.

Our main results suggested that a decline in health insurance coverage significantly accentuated the coal county effect. County health insurance rates captured the degree of health care coverage. Without any health insurance, patients might not be able to afford medical care, which may result in higher risks of dying from several chronic diseases [39]. In many coal-mining counties, the declining health insurance rate was a common problem, which reduced the affordability of health care services [40]. Since the demand for coal decreased in the United States, several coal companies declared bankruptcy and stopped contributing to the healthcare benefits for their retirees [16]. This might hurt health insurance coverage in coal counties. The uninsured can be expected to be more vulnerable to coal-related diseases that needed long-term medical care.

Coal-mining counties are often located in mountain areas and have limited access to health services such as fewer hospitals and physicians than the national average [2, 17]. The number of doctors reflected the community’s ability to detect diseases and provide long-term medical services. A shortage of physicians in Appalachian counties is associated with fewer appointment times [40]. For example, Wellmont Health system closed the only hospital in Lee County in 2013. After that closure, Lee County’s residents have to visit a hospital in a neighboring county for quick lab work or X-rays. Like Lee County, some poor Appalachian rural counties faced the problem of doctors leaving [41]. A survey by Huttlinger et al. [40] showed that many respondents in Appalachia had to wait up to 3 months for a doctor’s appointment due to the lack of specialty care providers. A longer waiting time may impede rural residents from seeking early treatment on their coal-related diseases and can increase the coal health effect. As several respiratory diseases related to coal exposure are often symptomless, regular screening tests by doctors can result in detection of these diseases at earlier stages when the treatment is more effective to prevent death. Without easy access to healthcare professionals, a patient has a lower chance of surviving as his or her disease progresses to a complicated form [9, 42].

Data limitation might explain the significantly positive coefficients of the number of hospital beds (Table 3). We do not know how many hospital beds are occupied for respiratory treatments, such as mechanical ventilation and oxygen therapy [43]. As a result, the relationship between respiratory mortality and number of hospital beds is unexpected. Another plausible explanation is reverse causality [44]. As the number of hospital beds represents the capacity of healthcare facilities [45], a county with a large number of hospital beds often has a big and more demanded hospital and may also be the result of high demands. Furthermore, patients from neighboring counties may travel to that hospital for treatments. These may all result in higher county mortality rates than neighboring counties.

Smoking and surface coal mining also contributed to the coal-county effect. Researchers observed much higher smoking rates [46] in central Appalachia than the national average [47]. Similar findings from previous literature also suggested that living in a county with surface coal mining was associated with more hospitalizations for asthma [48] and high mortality rates of chronic heart diseases [19].

As subgroup analyses revealed higher coal-county effects among male than female residents, we suspected that occupational health hazard from coal miners might partly drive the estimated coal-county effects. Similar findings were reported by Hendryx and Ahern [6], who found coal effect was higher for male than female residents and interpreted this phenomenon as a miner’s effect. For female residents in this study, living in a coal-mining county was not associated with a higher likelihood of dying from NMRD. A few previous studies found that female residents in coal-mining areas had a higher mortality risk than females in non-coal areas [3, 20]. Our study did not find a significantly positive coal county effect among the female subgroup, which might be due to ecological bias. According to Greenland and Morgenstern [49], ecological bias means “the failure of ecological- (aggregate-) level associations to properly reflect individual-level associations.” Although the aggregate effect on female subsample was not significant, living in a coal county might still increase the mortality risk for some female residents. Additionally, there are two potential reasons for our different results compared to previous studies. First, the ICD diagnosis codes used in our study were J00-J99 for NMRD. Previous studies focused on other health outcomes. Second, due to some unobserved factors, less healthy people may self-select to live in economically distressed counties. This lead to a concern of selection bias that the observed health disparity has no association with coal mining but with income. Previous researchers often compared mortality risks between coal-mining counties with non-coal-mining counties, which did not consider the issue of selection bias and income effects. Our study attempted to reduce the selection bias by using tobacco counties as the comparison group, given the similarity in economic condition between the coal-mining counties and tobacco counties.

In the second subgroup analysis, health effect of coal mining on the working-age residents was higher than that on retirement-age residents. Driven by the decline in health insurance coverage rate, an increase in coal county effect was observed for Russell County’s working population, but not for the retirement-age population in the same county, which reflected the crucial role of health insurance on the working population to reduce adverse health impact from coal production.

### Policy suggestions

Our findings assist health policymakers in identifying and choosing between alternative strategies when attempting to reduce elevated mortality rates in coal communities. First, affordability of health insurance challenges these coal communities due to declines in the coal industry during the past two decades [40], and thus, loss of jobs leads to loss of health benefits. Policy makers may consider expanding health insurance coverage by introducing low-cost health insurance plans and increasing diverse job opportunities. According to Perri [16], Congress reached a deal to provide a permanent $1.3 billion benefit for over 22,600 retired coal miners and their families, which may be helpful to increase health insurance coverage. Second, to address the shortage of doctors, healthcare facilities in coal-mining counties may consider collaborations with other healthcare facilities and increase incentives to recruit more healthcare professionals. Some rural counties may use telehealth [50], which allows patients to see a remote specialist by using video conferencing.

### Limitations

Common to previous studies, this study has several limitations. Although our analyses were based on individual-level data, the risk of ecological bias still existed. This problem happens when an inference is made for individuals based on aggregate data due to loss of intergroup variation in the distribution of other risk factors and effect modifiers. Although our regression analysis used individual death records and controlled individual-level covariates, there was a potential ecological bias when county-level covariates were introduced in the level-2 model. Particularly, the coal-county effect was an average health impact of living in a coal-mining county. Within each county, the coal health impact on each individual can be different. To assess ecological bias, future studies may consider analyzing the association at individual and different aggregation levels to see if there is a significant difference. If yes, appropriate control of individual-level covariates can reduce ecological bias [49].

One potential limitation of the statistical model is that it did not assess the spatial autocorrelation among counties. Previous spatial analyses found cancer mortality clustered in areas of heavy coal production [5, 24]. If NMRD mortality exhibits a positive spatial autocorrelation among counties, the estimated coefficients are still unbiased, but their standard errors will be underestimated. Future studies may incorporate spatial analyses to better understand the health effect of living in an adjacent coal county.

The model revealed the statistical association between coal-county residency and likelihoods of dying from NMRD, but not the causal relationship. As the coal-county effect is a parameter estimate associated with a coal-county dummy variable, it does not mean the main driving force for the significance of the parameter is coal mining. Lack of individual-level coal exposure and environmental measures made it difficult to identify the causal pathways linking coal mining and NMRD mortality. To establish a causal link, researchers need more sophisticated identification strategies, such as natural experiments, longitudinal data on both health, environment and coal mining.

Other important limitations are mainly associated with data availability. First, we used county of residence in the death records as a rough measurement of exposure to coal production, which did not capture the length of exposure. Second, separating coal miners’ occupational hazard from the community health effect is another common challenge in this field. The lack of separation may overestimate coal health effects on the general population. Since almost all coal miners are male, we estimated the coal-county effects based on female subgroup as a “second-best” strategy to exclude occupational health exposure. The results indicated that estimated coal-county effects should be lower if coal miners can be excluded from the sample.

## Conclusions

This study is a step forward in understanding the underlying factors that may be associated with a “coal-county effect” and helps identify factors that can be targeted to improve health in coal-mining counties. Using individual mortality data, we found a higher risk of dying from NMRD associated with living in a coal-mining county, but not with living in an adjacent county. This association was further accentuated by limited accessibility of health services--low health insurance coverage rates and lack of doctors.

This study contributes to the literature by showing the critical role of health access in reducing health disparities related to coal exposure, especially for the working population. Since coal-county effects may include occupational hazard, future research needs the occupation information to test whether or not living in a coal-mining county contributes to non-miners’ respiratory mortality. Depending on data availability, future research may also consider better measures of coal exposure such as distance from residence to the nearest coal mine site [51] and occupational histories [52]. The specific mechanism through which coal affects population health is not in the scope of this study. As previous studies suggested coal mining was a significant source of air pollutants [26, 53, 54], future studies may examine environmental factors such as particulate matter distribution and concentration near Appalachian coal-mining region to investigate the mechanism and associate relevant disease incidence.

## Supplementary information

**Additional file 1.** VIF test results of collinearity between socioeconomic and health access covariates.

**Additional file 2.** Average Marginal Effects on Probability of Dying from NMRD.

## Data Availability

The mortality data that support the findings of this study are available from the Virginia Department of Health Office of Vital Statistics, but restrictions apply to the availability of these data, which require IRB approval and are not publicly available. Data are however available from the authors upon reasonable request and with permission of the Virginia Department of Health.

## Abbreviations

ACS: American Community Survey
AHRF: Area Health Resources File
ARIES: Appalachian Research Initiative for Environmental Science
BRFSS: Behavioral Risk Factor Surveillance System
COPD: Chronic obstructive pulmonary disease
CPS: Current Population Survey
CWP: Coal Workers’ Pneumoconiosis
ICD: International Codes for Diseases
NMRD: Non-malignant respiratory diseases
SAHIE: Small Area Health Insurance Estimates
SES: Socioeconomic status

## Footnotes

1 Several previous studies identified elevated mortality rates in coal-mining areas but did not name their findings as “coal-county effects.

2 Health behavioral risk factors refer to risk behaviors that lead to poor health outcomes.

3 1 Counties in the metropolitan area: Rural-Urban Continuum codes 1–3 with the description of “Metro - Counties in metro areas”; 2) Non-metropolitan urban counties: Rural-Urban Continuum codes 4–7 with the description of “Nonmetro - Urban population of 2500 or more”; 3) Rural counties: Rural-Urban Continuum codes 8–9 with the description of “Nonmetro county completely rural or less than 2500 urban population.

4 In addition to 21 counties, there are three independent cities: Bristol City and Norton City in the coal region and Danville City in the tobacco region. So we have a total of 24 clusters.
