# Supplementary material for: Factors influencing high respiratory mortality in coal-mining counties: a repeated cross-sectional study
Source: BMC Public Health. 2019 Nov 8;19:1484. doi: 10.1186/s12889-019-7858-y (PMC6839055; doi:10.1186/s12889-019-7858-y)
Supplement: Supplementary file 1 — Additional file 1. VIF test results of collinearity between socioeconomic and health access covariates. [file 12889_2019_7858_MOESM1_ESM.docx]

**Additional file 1: VIF test results of collinearity between socioeconomic and health access covariates.**

|  | VIF | SQRT VIF | Tolerance |
| --- | --- | --- | --- |
| *SES* |  |  |  |
| $R_{unemploy}$ | 1.47 | 1.21 | 0.6786 |
| $Income$ | 2.16 | 1.47 | 0.4628 |
| $I_{metro}$ | 1.80 | 1.34 | 0.5550 |
| $I_{rural}$ | 1.93 | 1.39 | 0.5191 |
| *Health Access* |  |  |  |
| $Bed_{per1000}$ | 1.54 | 1.24 | 0.6507 |
| $Hcenter_{per1000}$ | 1.22 | 1.10 | 0.8206 |
| $Doctor_{per1000}$ | 1.39 | 1.18 | 0.7172 |
| $R_{insur}$ | 1.89 | 1.37 | 0.5291 |
