# Supplementary material for: Factors influencing high respiratory mortality in coal-mining counties: a repeated cross-sectional study
Source: BMC Public Health. 2019 Nov 8;19:1484. doi: 10.1186/s12889-019-7858-y (PMC6839055; doi:10.1186/s12889-019-7858-y)
Supplement: Supplementary file 2 — Additional file 2. Average Marginal Effects on Probability of Dying from NMRD. [file 12889_2019_7858_MOESM2_ESM.docx]

**Additional file 2: Average Marginal Effects on Probability of Dying from NMRD^a^**

|  | | (1) | | (2) | | (3) | | (4) | | (5) | |
| --- | --- | --- | --- | --- | --- | --- | --- | --- | --- | --- | --- |
|  | | General Model | | Model 1 | | Model 2 | | City Adjusted Model | | Scott Check Model | |
| *SES* | |  | |  | |  | |  | |  | |
| $R_{unemploy}$ | | 0.009^**^ | | 0.004 | |  | |  | |  | |
|  | | (2.02) | | (1.24) | |  | |  | |  | |
| $Income$ | | 0.003^**^ | | 0.002^*^ | |  | |  | |  | |
|  | | (2.46) | | (1.77) | |  | |  | |  | |
| $I_{metro}$ | | 0.021^**^ | | 0.019^*^ | |  | |  | |  | |
|  | | (2.08) | | (1.88) | |  | |  | |  | |
| $I_{rural}$ | | 0.014^**^ | | 0.017^**^ | |  | |  | |  | |
|  | | (2.22) | | (2.21) | |  | |  | |  | |
| *Health Access* | |  | |  | |  | |  | |  | |
| $Bed_{per1000}$ | | 0.007^***^ | | 0.007^***^ | | 0.005^***^ | | 0.005^***^ | | 0.005^***^ | |
|  | | (8.72) | | (8.26) | | (6.98) | | (7.47) | | (8.08) | |
| $Hcenter_{per1000}$ | | -0.114^***^ | | -0.130^***^ | | -0.132^***^ | | -0.132^***^ | | -0.126^***^ | |
|  | | (-9.01) | | (-8.90) | | (-7.40) | | (-7.40) | | (-9.68) | |
| $Doctor_{per1000}$ | | -0.005 | | -0.009^**^ | | -0.008^***^ | | -0.008^***^ | | -0.008^***^ | |
|  | | (-1.15) | | (-2.01) | | (-4.14) | | (-4.14) | | (-4.45) | |
| $R_{insur}$ | | -0.016^***^ | | -0.015^***^ | | -0.014^***^ | | -0.014^***^ | | -0.014^***^ | |
|  | | (-5.16) | | (-4.85) | | (-4.41) | | (-4.78) | | (-3.88) | |
| *Risk Factor* | |  | |  | |  | |  | |  | |
| $R_{obesity}$ | | -0.002 | | -0.002 | | -0.0004 | | -0.0004 | | -0.002 | |
|  | | (-0.71) | | (-0.80) | | (-0.21) | | (-0.21) | | (-1.14) | |
| $R_{inactivity}$ | | -0.002^**^ | | -0.002^**^ | | -0.002^*^ | | -0.002^*^ | | -0.001 | |
|  | | (-2.14) | | (-2.01) | | (-1.75) | | (-1.81) | | (-1.55) | |
| $R_{smoking}$ | | 0.005^**^ | | 0.003 | | 0.004^***^ | | 0.004^***^ | | 0.004^***^ | |
|  | | (2.53) | | (1.44) | | (2.87) | | (2.84) | | (2.75) | |
| *Coal-Related Variables* | | | |  | |  | |  | |  | |
| $Surface\%$ | | 0.001^***^ | | 0.001^***^ | | 0.0005^***^ | | 0.0005^***^ | | 0.0005^***^ | |
|  | | (9.11) | | (9.68) | | (4.70) | | (4.71) | | (4.59) | |
| $d_{incoal}$ | | 0.029^***^ | | 0.018^**^ | | 0.014^**^ | | 0.014^**^ | | 0.018^**^ | |
|  | | (3.12) | | (2.53) | | (2.07) | | (2.03) | | (2.60) | |
| $d_{adjcoal}$ | | 0.002 | | -0.004 | | -0.008 | | -0.008 | | -0.001 | |
|  | | (0.19) | | (-0.58) | | (-1.04) | | (-1.15) | | (-0.14) | |
| *Control Variables* | | | |  | |  | |  | |  | |
| ${Race}_{black}$ | | -0.044^***^ | | -0.044^***^ | | -0.044^***^ | | -0.044^***^ | | -0.044^***^ | |
|  | | (-11.67) | | (-11.69) | | (-11.63) | | (-12.15) | | (-11.72) | |
| ${Race}_{other}$ | | -0.062 | | -0.063 | | -0.062 | | -0.062 | | -0.062 | |
|  | | (-1.32) | | (-1.32) | | (-1.32) | | (-1.29) | | (-1.31) | |
| ${Sex}_{female}$ | | -0.015^***^ | | -0.015^***^ | | -0.015^***^ | | -0.015^***^ | | -0.015^***^ | |
|  | | (-3.29) | | (-3.30) | | (-3.31) | | (-3.39) | | (-3.31) | |
|  | |  | |  | |  | |  | |  | |
|  | | (1) | | (2) | | (3) | | (4) | | (5) | |
|  | | General Model | | Model 1 | | Model 2 | | City Adjusted Model | | Scott Check Model | |
|  | |  | |  | |  | |  | |  | |
| Single | | 0.003 | | 0.003 | | 0.003 | | 0.003 | | 0.003 | |
|  | | (0.72) | | (0.72) | | (0.72) | | (0.78) | | (0.70) | |
| Married | | -0.008^*^ | | -0.008^*^ | | -0.008^*^ | | -0.008^**^ | | -0.008^*^ | |
|  | | (-1.92) | | (-1.93) | | (-1.91) | | (-2.17) | | (-1.92) | |
| Divorced | | -0.001 | | -0.001 | | -0.0005 | | -0.0005 | | -0.001 | |
|  | | (-0.09) | | (-0.10) | | (-0.07) | | (-0.07) | | (-0.08) | |
| Age | | 0.001^***^ | | 0.001^***^ | | 0.001^***^ | | 0.001^***^ | | 0.001^***^ | |
|  | | (7.15) | | (7.21) | | (7.22) | | (7.14) | | (7.24) | |
| Education year | | -0.005^***^ | | -0.005^***^ | | -0.005^***^ | | -0.005^***^ | | -0.005^***^ | |
|  | | (-11.96) | | (-11.96) | | (-11.95) | | (-11.76) | | (-11.98) | |

*Note:* z test statistic in parentheses ^*^ *p* < .1, ^**^ *p* < .05, ^***^ *p* < .01.

^a^ Year dummies are controlled, but their marginal effects are not reported in additional table 2. County-level variables’ marginal effects are the average marginal effects of coal-mining residents. Individual control variables’ marginal effects are the average marginal effects of the whole sample.
